# Ablative Five-Fraction Stereotactic Radiotherapy in Elderly Patients with Pancreatic Cancer: A Single Institution Experience

**DOI:** 10.3390/jcm13247739

**Published:** 2024-12-18

**Authors:** Michele Fiore, Gian Marco Petrianni, Gabriele D’Ercole, Elena Onorati, Pasquale Trecca, Rita Alaimo, Daniele Carlotti, Edy Ippolito, Rolando Maria D’Angelillo, Sara Ramella

**Affiliations:** 1 Research Unit of Radiation Oncology, Department of Medicine and Surgery, Università Campus Bio-Medico di Roma, Via Alvaro del Portillo, 21-00128 Rome, Italy; m.fiore@policlinicocampus.it (M.F.); s.ramella@policlinicocampus.it (S.R.); 2 Operative Research Unit of Radiation Oncology, Fondazione Policlinico Universitario Campus Bio-Medico, Via Alvaro del Portillo, 200-00128 Rome, Italy; 3 Radiation Oncology, Tor Vergata University, Viale Oxford, 81-00133 Rome, Italy

**Keywords:** SBRT, pancreatic cancer, elderly patients

## Abstract

**Background:** Pancreatic cancer, with its poor prognosis, is frequently diagnosed in elderly patients who may be ineligible for surgery or chemoradiation due to age or comorbidities. Stereotactic body radiotherapy (SBRT) offers a targeted approach by delivering precise, high-dose radiation to a limited volume in few fractions. This study aims to evaluate the efficacy and safety of SBRT in elderly pancreatic cancer patients. **Methods:** Patients with pancreatic cancer received SBRT using Volumetric Modulated Arc Therapy in schedules of five dose fractions tailored to the tolerance of adjacent tissues. Progression-free survival (PFS), local progression-free survival (LPFS), metastasis-free survival (MFS) and overall survival (OS) were analysed. Toxicities were graded according to the Common Terminology Criteria for Adverse Events (CTCAE) v5.0. **Results:** Median follow-up was 9.5 months (range, 4–37.2 months). The 1-year, 2-year and 3-year LPFS rates were 88%, 73% and 54%, respectively (median not reached). Median PFS, MFS and OS were 29.6 months, 32.6 months, and 24.1 months, respectively. SBRT was well tolerated with acute and late toxicity rates of 6% and 9%, respectively. **Conclusionsd:** SBRT appears to be an effective and safe option for elderly pancreatic cancer patients, achieving high local control with minimal toxicity.

## 1. Introduction

Pancreatic cancer is a major challenge in oncology, with a poor prognosis and increasing incidence. In the United States, the projected number of new cases and deaths in 2024 (66,440 and 51,750, respectively) underscores the urgency of addressing this malignancy [1]. Aging demographics have contributed to a significant increase in diagnoses in older patients, who often face limited treatment options due to comorbidities. Despite advances in medical care, survival rates remain woefully low, which calls for a deeper understanding of the factors that influence outcomes. Pancreatic cancer mortality patterns differ based on age and gender. Among individuals aged 65–79, it is the third most common cause of cancer death in both men and women, after colorectal and lung cancer in men and lung and breast cancer in women. However, in people aged 80 years and over, pancreatic cancer falls to fifth place in men, after lung, prostate, colorectal and bladder cancers, and to fourth place in women, after lung, breast and colorectal cancers [2]. These differences highlight the need for tailored interventions to address the burden of pancreatic cancer in specific population groups.

One of the major concerns in the management of this disease is the early detection in high-risk individuals and the timely diagnosis of patients presenting with suspected symptoms. Early detection is critical not only to improve clinical outcomes but also to avoid unnecessary interventions in patients who may not require aggressive treatment. Identifying effective strategies to diagnose pancreatic cancer at an earlier stage has the potential to significantly improve prognosis and optimise patient care. Recent advances have highlighted the importance of targeted screening in high-risk populations and the role of novel biomarkers in improving diagnostic accuracy. These strategies aim to bridge the gap between symptom onset and diagnosis, allowing for timely therapeutic intervention [3].

The paradigm of precision oncology, epitomised by multidisciplinary tumour board meetings, is central to the design of personalised treatment plans. This collaborative approach, integrating expertise from multiple specialties, facilitates accurate staging and precise diagnosis, thereby guiding optimal therapeutic strategies tailored to individual patient needs [4]. Adopting this patient-centred model aligns clinical practice with the evolving landscape of personalised medicine [5].

Radiotherapy plays a pivotal role in the treatment of pancreatic cancer in various settings, including neoadjuvant, adjuvant, curative and palliative. It is commonly delivered using conventional or hypofractionated schedules and is often given in combination with chemotherapy [6,7,8]. Stereotactic body radiotherapy (SBRT) is emerging as a valuable therapeutic option for elderly patients with pancreatic cancer, particularly those with impaired performance status and logistical barriers [9]. Delivered with high precision by linear accelerators or dedicated machines, SBRT delivers higher doses in fewer fractions to a precisely defined target volume, tailored to minimise damage to adjacent organs at risk [10]. Therapeutic goals include both symptom control and prognostic improvement. However, ensuring the accuracy of SBRT requires refined protocols across the clinical pathway, including multidisciplinary consultation, rigorous staging, optimal treatment selection and meticulous follow-up [11].

This study focuses on evaluating the local control rates and safety profile of SBRT in elderly patients with pancreatic cancer. By investigating these aspects, we aim to provide further data on the efficacy and safety of SBRT in this population, ultimately leading to improved treatment decisions for this challenging patient cohort.

## 2. Materials and Methods

In this retrospective monocentric study, we enrolled a cohort of elderly patients diagnosed with pancreatic cancer who received SBRT for a primary tumour. All participants had histologically confirmed pancreatic cancer and were considered unresectable for surgery due to tumour characteristics, predominantly vascular–tumour connections, and patient-related factors such as advanced age and comorbidities. Patients signed an informed consent for their clinical data to be used for scientific purposes.

All patients underwent a thorough pre-treatment staging including endoscopic ultrasound (EUS) with fine needle aspiration biopsy, a multilayer computed tomography (CT) scan of the chest, abdomen and pelvis using intravenous contrast and a 18F-FDG PET-CT scan. Patients who were jaundiced prior to treatment underwent endoscopic biliary stenting. CT and 18F-FDG PET-CT scans were used to obtain a precise delineation of the clinical target volume (CTV) and to define the organs at risk (OAR).

All patients who were candidates for SBRT underwent simulation using a 3 mm-thick CT scan. Patients were positioned appropriately using immobilisation systems, followed by intravenous contrast administration. Simulation CT scans were performed using breathing control techniques, including Deep Inspiration Breath Hold (DIBH), Deep Expiration Breath Hold (DEBH) and four-dimensional CT (4D-CT), to ensure optimal visualisation of tumour volumes while minimising motion artefacts. The internal target volume (ITV) was created from the available 4D-CT scan by contouring the tumour on multiple phases of respiration. A PTV including the ITV with a circumferential margin of 5 mm was used.

Patients were treated using the Volumetric Modulated Arc Therapy (VMAT) technique. Four different schedules were used to deliver the dose to the PTV: 30 Gy in five fractions (600 cGy/day), 35 Gy in five fractions (700 cGy/day), 40 Gy in five fractions (800 cGy/day) and 45 Gy in five fractions (900 cGy/day), taking into account 80% inhomogeneity to the isocentre dose. Table 1 details the dose constraints for OARs.

SBRT treatments were delivered using linear accelerators, including TrueBeam^®^ (Varian Medical Systems, Inc., Palo Alto, CA, USA, Elekta Inc., Stockholm, Sweden) and Versa HD^®^ (Elekta Inc., Stockholm, Sweden), with image-guided radiotherapy (IGRT) facilitated by daily cone beam CT imaging.

All study sample characteristics were summarised with descriptive statistics. The primary endpoint of this study was the local control as assessed by a CT scan. In addition, the safety profile of the SBRT approach was evaluated by monitoring acute and late toxicities using the National Cancer Institute Common Terminology Criteria for Adverse Events (NCI CTCAE) version 5.0 scale.

Data were retrospectively collected and statistical analysis was performed for endpoints, including progression-free survival (PFS), local progression-free survival (LPFS), metastasis-free survival (MFS) and overall survival (OS), using the Statistical Package for Social Sciences, version 27 (SPSS Inc., Chicago, IL, USA). Progression-free survival was determined from the start of treatment until the observation of progression/recurrence or until the last follow-up if no event was observed. Overall survival was determined from the date of histological diagnosis. PFS and OS curves were calculated using the Kaplan–Meier method. The analysis included the chi-square test of independence to examine relationships between categorical variables. A significance level (α) of 0.05 was used to determine statistical significance.

## 3. Results

### 3.1. Patient and Treatment Characteristics

The population study included thirty-three patients (11 males and 22 females) with a mean age of 77 years (range, 65–90) who were treated at our institution between July 2018 and May 2024. Most of the patients had good performance status (ECOG score 0–1) and only one patient had an ECOG score of 2. All cases were discussed in the institutional multidisciplinary tumour board. After clinical and radiological evaluation, all patients were clinically staged according to the American Joint Committee on Cancer (AJCC) 8th edition staging system. Fourteen patients (42.3%) had stage I disease (three stage IA and eleven stage IB), five patients (15.2%) had stage II disease (one stage IIA and four stage IIB), eight patients (24.3%) had stage III disease and six patients (18.2%) had metastatic disease at diagnosis.

Thirty-one patients had a histologically proven diagnosis of pancreatic cancer (26 adenocarcinoma and five other histology), while two patients were not histologically typed due to their clinical status and the procedural risk, so the diagnosis was based on imaging.

The mean value of serum Ca 19-9 level at the time of diagnosis was 1117 U/ml (range 1–9532 U/mL). Regarding tumour location, most tumours (27 of 33, 81.8%) were located in the head of the pancreas, while the remaining (six of thirty-three, 18.2%) were located in the body and/or tail of the pancreas.

In terms of surgical eligibility, twenty-two patients (66.7%) had tumours that were considered surgically resectable but were not amenable to surgery due to age, comorbidities and general condition (18 patients) or because they were metastatic at diagnosis (four patients). Eight patients (24.2%) had disease classified as borderline resectable, two of whom were metastatic at diagnosis. Three patients (9.1%) had locally advanced tumours that were considered unresectable. Patients’ characteristics are summarised in Table 2.

Treatment schedules included 30 Gy in five fractions for twenty-three patients (69.7%), 35 Gy in five fractions for eight patients (24.3%), 40 Gy in five fractions for one patient (3%) and 45 Gy in five fractions for one patient (3%). For each schedule, we calculated the biologically effective dose (BED10), which was 48 Gy, 59.5 Gy, 72 Gy and 85.5 Gy, respectively, given that these were stereotactic treatments and assuming an alpha/beta ratio of 10 for the tumour.

Fourteen patients (42.4%), including the six patients with metastatic disease at diagnosis, received chemotherapy prior to SBRT. Of these, six metastatic patients (18.2%) continued chemotherapy immediately after SBRT. Of the remaining eight patients, four resumed chemotherapy 6 months after completion of SBRT due to disease progression. Chemotherapy included various regimens such as gemcitabine–abraxane, FOLFIRINOX, and gemcitabine as monotherapy, and was selected according to the patients’ clinical conditions. Four patients (12%) underwent surgery after SBRT, and two of whom had resectable disease but were initially considered inoperable due to clinical conditions. One had a borderline resectable disease, and one had an unresectable locally advanced tumour. These data are summarised in Table 3.

### 3.2. Treatment Efficacy and Outcomes

All patients completed SBRT. Twenty-seven patients (81.8%) were evaluable for tumour response according to RECIST criteria at 4 months post-treatment, while six patients (18.2%) were lost to follow-up due to poor overall health. At the first follow-up, seventeen patients (63%) had stable disease, seven patients (25.9%) had a partial response, and three patients (11.1%) experienced disease progression. The initial mode of disease progression was local recurrence in five patients, distant metastasis in six patients and both local and distant failure in two patients.

In particular, regarding the patterns of recurrence in patients with progression, our analysis showed that among those with local recurrence, four patients had an increase in the size of the primary tumour, while three patients had progression in regional lymph nodes. For distant metastases, five patients developed liver metastases, two had peritoneal metastases and one had both lung and peritoneal metastases.

In patients with local progression, the mean prescribed radiotherapy dose was 32 Gy (range, 30–45 Gy). For all patients, the mean biologically effective dose (BED10) was 52.7 Gy (range, 48–85.5 Gy). For those with local disease progression, the mean BED10 was 51.4 Gy (range, 48–85.5 Gy).

The relationship between clinical and treatment-related factors and the occurrence of disease progression was evaluated. The results are summarised in Table 4. Of the variables analysed, most did not show a statistically significant association with progression. However, tumour site and radiotherapy dose emerged as significant predictors of progression. In particular, tumour location in the head of the pancreas was found to correlate with an increased likelihood of progression (*p* = 0.04). In addition, a total radiotherapy dose of 30 Gy or lower was associated with a higher risk of progression (*p* = 0.03).

In our cohort, six patients (18%) experienced pain prior to SBRT, as assessed by the Numerical Rating Scale (NRS) scoring system. After treatment, all patients experienced pain relief. Complete pain relief was achieved in three patients, allowing discontinuation of analgesics. The analgesic dose was reduced by 50% in two patients and by 20% in one patient.

The median follow-up for the whole population was 9.5 months (range, 4–37.2 months). Table 5 details the survival results. 

**Figure 1:**
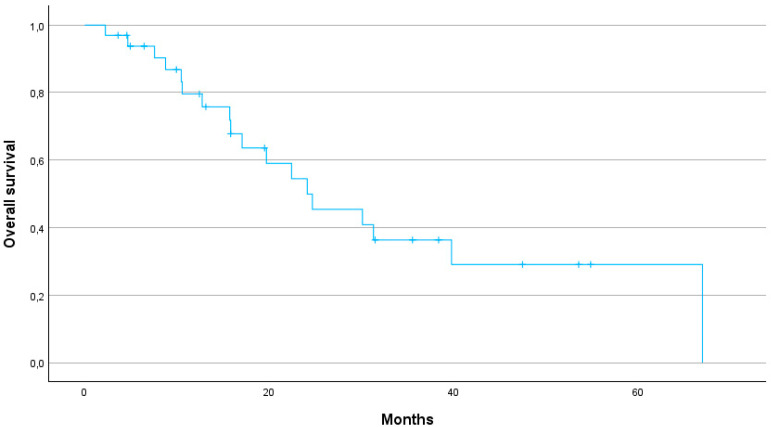
Kaplan–Meier curve for Overall survival.

The median OS for all patients was 24.1 months (95% CI: 23.6–43). The 1-year, 2-year and 3-year OS rates were 75.8, 50 and 29%, respectively (Figure 1).

**Figure 2:**
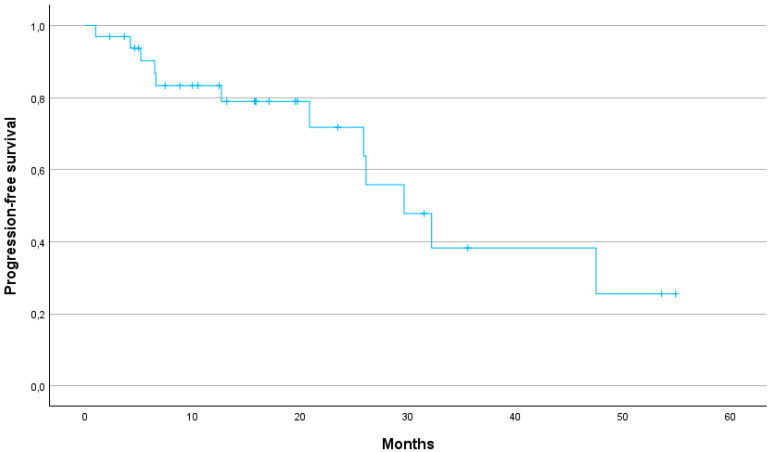
Kaplan–Meier curve for progression-free survival.

**Figure 3:**
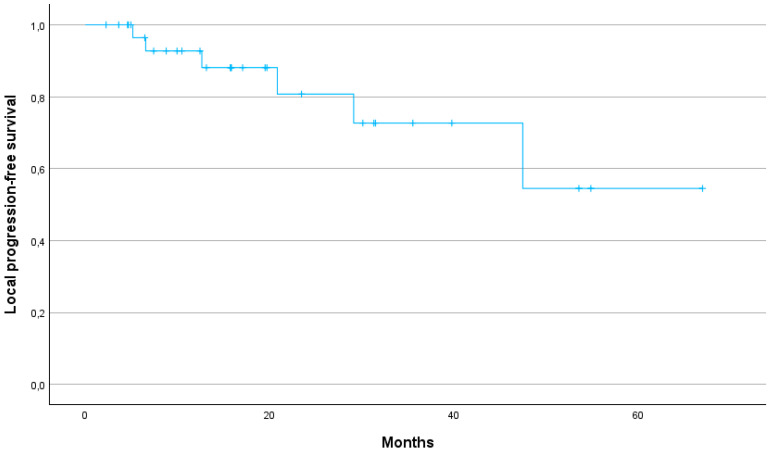
Kaplan–Meier curve for local progression-free survival.

**Figure 4:**
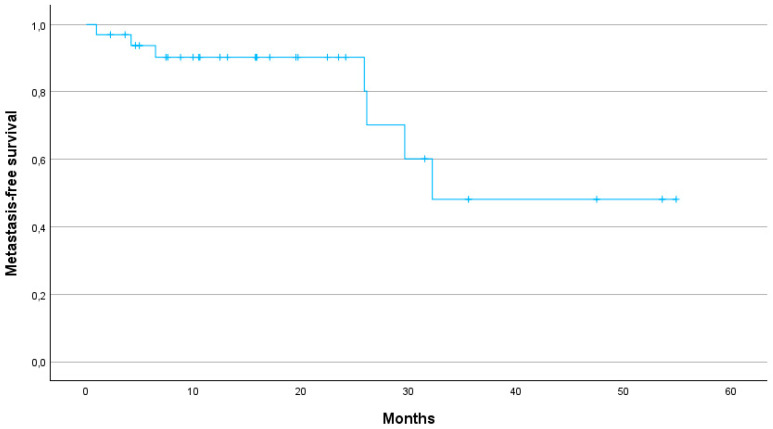
Kaplan–Meier curve for metastasis progression-free survival.

The median PFS was 29.6 months (95% CI: 24.2–40.7). The 1-year, 2-year and 3-year PFS were 79, 64 and 25%, respectively (Figure 2). The 1-year, 2-year and 3-year LPFS were 88, 73 and 54%, respectively (median not reached) (Figure 3). The 1-year, 2-year and 3-year MFS were 90, 80 and 48%, respectively, with a median MFS of 32.2 months (95% CI: 29.8–47.9) (Figure 4).

**Figure 5:**
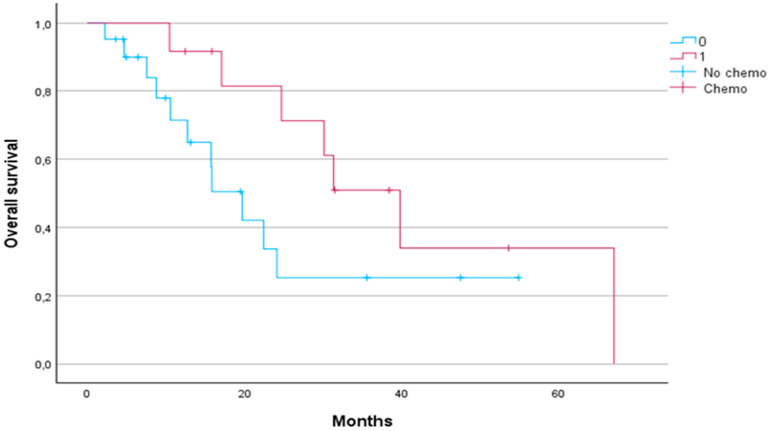
Kaplan–Meier curve for overall survival comparing the group of patients who received chemotherapy (red line) and the group who did not receive chemotherapy (blue line).

Patients who received chemotherapy had a significantly longer median OS compared to patients who did not receive chemotherapy (31.33 months vs. 15.8 months, respectively, *p* < 0.05) (Figure 5).

There was no significant difference in either PFS or LPFS between patients who received chemotherapy and those who did not (median PFS 19.2 vs. 28.4 months, *p* = 0.4; median LPFS 19.2 vs. 28.4 months, *p* = 0.4).

Additionally, we analysed survival outcomes according to the type of chemotherapy administered (gemcitabine-based regimens versus FOLFIRINOX) and found no significant differences in terms of OS (*p* = 0.6), PFS (*p* = 0.3), LPFS (*p* = 0.2) or MFS (*p* = 0.8) between groups.

Several variables were analysed at univariate analysis, such as age over 75 years, age over 80 years, sex, tumour location, tumour size, lymph node status, chemotherapy administration and radiotherapy dose. In both univariate and multivariate analysis, no variables correlated with either clinical response to SBRT or disease progression at six months after the end of radiotherapy.

### 3.3. Treatment-Related Toxicity

In terms of safety, SBRT was well tolerated. Two acute toxicities (6%) were observed, while only three cases (9%) of late toxicities were reported. Regarding acute toxicities, one patient experienced radiation-induced gastroduodenitis (grade 3 according to CTCAE v 5.0) 30 days after completion of radiotherapy, requiring hospitalisation and steroid therapy. The other patient developed acute cholangitis (grade 3) 30 days after completion of treatment due to obstruction of the biliary prosthesis, requiring hospitalisation and CPRE.

In terms of late toxicities, one patient had duodenal stenosis (grade 3) six months after the end of the radiotherapy, for which hospitalisation and duodenal prosthesis placement were required. Another patient had a duodenal ulcer (grade 2) due to radiation-induced duodenitis, which was treated with appropriate medical therapy. The other patient, who underwent a duodenocephalopancreasectomy after the SBRT, had a pancreatic fistula (grade 2) six months after the end of the radiotherapy treatment, which required hospitalisation and was treated conservatively with antibiotic therapy.

## 4. Discussion

Our study supports the use of SBRT as an effective treatment for pancreatic cancer and provides insight into disease control, survival outcomes and safety profiles in an elderly patient cohort. With a median age of 77 years, our patients were not candidates for surgery or chemoradiation due to age and comorbidities. Our findings are consistent with current evidence and highlight the role of SBRT in maintaining local control and prolonging survival with a manageable toxicity profile, even in elderly and medically complex patients.

Several studies have demonstrated the potential benefits of SBRT over conventional radiotherapy in terms of OS, local control (LC) and toxicity profiles. The meta-analysis by Tchelebi et al. highlights a significant improvement in 2-year OS (27% vs. 14%) and a more favourable acute toxicity profile (6% vs. 38%) when comparing SBRT with conventional fractionated radiotherapy in LAPC patients [13]. In our cohort, 1-, 2- and 3-year OS rates were 75.8%, 50%, and 29%, respectively, further supporting the durability of SBRT in achieving prolonged survival. Our results are in line with previous studies suggesting that SBRT offers significant survival benefits over conventional radiotherapy. In particular, the median OS of 24.1 months in our cohort is consistent with the upper range reported in the literature, where SBRT-treated pancreatic cancer patients demonstrate an OS of 12 to 24 months [7]. Early studies using one to three fractions of SBRT achieved high LC rates, but at the cost of severe late gastrointestinal toxicity [14,15,16]. In contrast, more recent SBRT protocols using five fractions have achieved a better balance between maintaining high LC and reducing toxicity. This approach has been underlined by a recent systematic review of 14 prospective studies in which SBRT demonstrated favourable LC rates between 78% and 90% at one year [17]. In our study, LC was similarly promising with a 1-year LC rate of 88%, a 2-year LC rate of 73% and a 3-year LC rate of 54%. Our study thus demonstrates that SBRT can provide high LC even in patients for whom surgical options are limited due to age or comorbidities. Furthermore, the 1-year MFS of 90% and the median MFS of 32.2 months suggest that SBRT can effectively delay disease progression, contributing to prolonged survival.

In our cohort, tumour location in the head of the pancreas was found to correlate with an increased likelihood of progression (*p* = 0.04). This finding may be due to the anatomical proximity of the pancreatic head to critical structures such as the duodenum. This proximity often limits the ability to safely deliver higher doses of radiotherapy due to the increased risk of gastrointestinal toxicity. As a result, efforts to minimise duodenal toxicity may require dose reductions, potentially compromising local tumour control and increasing the risk of disease progression. The observed association between a total radiotherapy dose of 30 Gy or less and a higher risk of progression (*p* = 0.03) further highlights the critical role of dose escalation in improving treatment outcomes. However, achieving this in pancreatic head tumours presents a unique challenge, as higher doses may increase the risk of toxicity to adjacent gastrointestinal structures. These findings highlight the need for careful treatment planning that balances the benefits of dose escalation with the potential risks of toxicity, particularly in patients with pancreatic head tumours. Advanced radiotherapy techniques, such as adaptive planning and image-guided radiotherapy, may play a critical role in overcoming these challenges to optimise local control while minimising treatment-related toxicity. Our findings suggest that local control strategies may need to be complemented by systemic treatments to address the risk of distant metastases. Further studies are warranted to better understand these patterns of progression and to evaluate how SBRT, in combination with other therapeutic modalities, may contribute to mitigate these outcomes.

Our study highlights the value of SBRT in patients who are ineligible for surgery due to age or comorbidities. In our cohort, 66.7% of patients were considered unresectable due to these factors, and only four patients underwent surgery after SBRT. Notably, our results are consistent with the real-world analysis by Arcelli et al. [18], which demonstrated prolonged LC with SBRT compared to chemotherapy alone and showed superior LC with SBRT compared to chemoradiation in multivariable analyses (HR: 0.46, 95% CI 0.25–0.83; *p* = 0.011). For patients who are not candidates for surgery, SBRT is therefore a valuable alternative, offering disease control and symptom relief comparable to, or potentially better than, other non-surgical options.

Our results suggest that SBRT may be synergistic with chemotherapy. Patients who received chemotherapy before or after SBRT had a significantly longer median OS (31.3 months) compared to those who did not receive chemotherapy (15.8 months, *p* < 0.05). Although no significant differences in PFS or LPFS were observed based on chemotherapy administration, the survival benefit underlines the value of a multimodality approach, particularly given the heterogeneous nature of pancreatic cancer and its variable response to treatment. This finding is in line with trends in recent studies showing the increasing use of FOLFIRINOX in combination with SBRT, resulting in better survival rates than gemcitabine-based regimens alone [7].

In addition to survival and LC benefits, SBRT offers significant symptomatic relief for patients with pancreatic cancer. Studies by Buwenge et al. [9] and Vornhülz et al. [19] highlight the role of SBRT in managing pain and improving quality of life, with response rates as high as 84.9% for pain relief and reduction of symptoms such as jaundice and nausea. Our cohort demonstrated stable disease in 63% of patients, partial response in 25.9% and progressive disease in only 11.1% at first follow-up, suggesting that SBRT not only controls the tumour but also helps to stabilise symptoms, contributing to improved quality of life. All patients who had pain at the time of SBRT experienced a reduction in their symptoms.

The safety of SBRT in our study population was confirmed by low rates of both acute and late toxicities. Acute grade ≥3 toxicity was limited to two cases (6%), and late grade ≥ 2 toxicity was observed in only three patients (9%). These rates are consistent with the toxicity profiles reported in meta-analyses by Shouman et al. [17] and Petrelli et al. [20], where grade ≥ 3 toxicities ranged from 0% to 34%, supporting the favourable safety profile of SBRT. The nature of the toxicities in our study, which included gastroduodenitis, duodenal stenosis and duodenal ulceration, indicates the potential gastrointestinal complications associated with pancreatic SBRT. However, the overall low toxicity rates combined with a favourable LC confirm that SBRT is a well-tolerated treatment option, even in elderly patients or those with comorbidities that preclude more aggressive treatments.

This study has several strengths that provide valuable insights into the use of SBRT in an older patient population with pancreatic cancer. One of the most important strengths is the study’s focus on a real-world cohort, including patients who were often ineligible for surgery due to age or comorbidities. This makes our findings particularly relevant to similar high-risk patient groups that may not be well represented in clinical trials. Another strength is the uniform approach to SBRT treatment planning within our institution, which ensures consistency in dose delivery and minimises variability in therapeutic outcomes. In addition, the detailed analysis of clinical outcomes provides a comprehensive view of the efficacy of SBRT in this population.

However, our study has limitations. The sample size is relatively small and the follow-up period is limited, which may limit the evaluation of long-term outcomes, particularly in assessing the durability of local control and potential late toxicities. These limitations highlight the need for larger, multi-center studies with longer follow-up to confirm the durability of local control and to comprehensively assess potential long-term side effects. In addition, the retrospective nature of the study and the variety of chemotherapy regimens used limit the ability to standardise treatment effects and may introduce selection bias. Furthermore, specific data on other aspects of symptom relief, such as overall quality of life or additional symptom measures, were not comprehensively collected in this current study. This underlines the need for future research to include detailed quality of life assessments to provide a more comprehensive assessment of the wider benefits of SBRT.

## 5. Conclusions

Our results support the growing evidence for SBRT as a viable, effective and tolerable treatment option for pancreatic cancer, particularly in elderly or medically frail patients. With favourable survival rates, high local control and manageable toxicity profiles, SBRT stands out as an important therapeutic strategy. Given the improved outcomes associated with chemotherapy, especially in medically fit patients, SBRT in combination with chemotherapy should be considered for optimal management of pancreatic cancer.

## Figures and Tables

**Table 1:** OARs constraints for five fractions SBRT [12].

Duodenum	Dmax (0.1 cm^3^) < 35 Gy
Kidneys	V_17.5_ < 200 cm^3^
Liver	V_15_ < 700 cm^3^
Stomach	Dmax (0.1 cm^3^) < 35 Gy
Small Bowel	Dmax (0.5 cm^3^) < 35 Gy

**Table 2:** Patients’ characteristics.

Characteristics	No of Patients (n = 33)	%
**Age**		
Mean	77	
Range	65–90	
**Sex**		
Male	11	33.3
Female	22	66.7
**Disease stage**		
Stage I	14	42.3
Stage II	5	15.2
Stage III	8	24.3
Stage IV	6	18.2
**Ca 19.9 at diagnosis, U/mL**		
Median	1117	
Range	1–9532	
**Tumour localisation**		
Head	27	81.8
Body/Tail	6	18.2
**Resectability status**		
Resectable	22	66.7
Borderline resectable	8	24.2
Locally advancedunresectable	3	9.1

**Table 3:** Summary of treatment protocols and dosing details.

Treatment		No of Patients	%
**SBRT Doses**	**BED10 (Gy)**		
30 Gy (600 cGy/fr)	48	23	69.7
35 Gy (700 cGy/fr)	59.5	8	24.3
40 Gy (800 cGy/fr)	72	1	3
45 Gy (900 cGy/fr)	85.5	1	3
**Chemotherapy before SBRT**		14	42.4
Gemcitabine-Abraxane		7	21.2
FOLFIRINOX		4	12.2
Gemcitabine		3	9
**Chemotherapy after SBRT**		6	18.2
**Surgery after SBRT**		4	12.2

**Table 4:** Relationship between clinical and treatment-related variables and disease progression.

		No Disease Progression		Disease Progression		*P* *χ^2^ Test*
		N	%	N	%	
**ECOG-PS** **at diagnosis**	0	11	33.3	9	27.3	0.39
	1	9	27.3	3	9.1	
	2	1	3.0	0	0	
**Age**	<75 years	9	27.3	5	15.1	0.94
	>75 years	12	36.4	7	21.2	
**Tumour localisation**	Head	15	45.4	12	36.4	0.04
	Body/Tail	6	18.2	0	0	
**Tumour dimension**	<3 cm	8	24.3	5	15.1	0.84
	≥3 cm	13	39.4	7	21.2	
**Resectability status**	Resectable	14	42.4	8	24.3	0.99
	Borderline/Unresectable	7	21.2	4	12.1	
**Prescribed dose**	≤30 Gy	12	36.4	11	33.3	0.03
	>30 Gy	9	27.3	1	3.0	

**Table 5:** Survival outcomes.

	Median (Months)	1 Year (%)	2 Years (%)	3 Years (%)
**OS**	24.1	75.8	50	29
**PFS**	29.6	79	64	25
**LPFS**	-	88	73	54
**MFS**	32.2	90	80	48

OS: Overall survival; PFS: Progression-free survival; LPFS: Local progressio-free survival; MFS: Metastasis-free survival.

## Data Availability

The data presented in this study are available from the corresponding author upon reasonable request.
